# Epicardial adipose tissue predicts incident cardiovascular disease and mortality in patients with type 2 diabetes

**DOI:** 10.1186/s12933-019-0917-y

**Published:** 2019-08-30

**Authors:** Regitse H. Christensen, Bernt Johan von Scholten, Christian S. Hansen, Magnus T. Jensen, Tina Vilsbøll, Peter Rossing, Peter G. Jørgensen

**Affiliations:** 1 Center for Inflammation and Metabolism/Center for Physical Activity Research, Rigshospitalet, Copenhagen, Denmark; 2 Steno Diabetes Center Copenhagen, Gentofte, Denmark; 3 Department of Cardiology, Glostrup-Rigshospitalet, Glostrup, Denmark; 4 Department of Cardiology, Herlev-Gentofte Hospital, Hellerup, Denmark; 5 Department of Clinical Medicine, Faculty of Health and Medical Sciences, University of Copenhagen, Copenhagen, Denmark

**Keywords:** Epicardial adipose tissue, Pericardial adipose tissue, Cardiovascular disease, Risk prediction

## Abstract

**Background:**

Cardiac fat is a cardiovascular biomarker but its importance in patients with type 2 diabetes is not clear. The aim was to evaluate the predictive potential of epicardial (EAT), pericardial (PAT) and total cardiac (CAT) fat in type 2 diabetes and elucidate sex differences.

**Methods:**

EAT and PAT were measured by echocardiography in 1030 patients with type 2 diabetes. Follow-up was performed through national registries. The end-point was the composite of incident cardiovascular disease (CVD) and all-cause mortality. Analyses were unadjusted (model 1), adjusted for age and sex (model 2), plus systolic blood pressure, body mass index (BMI), low-density lipoprotein (LDL), smoking, diabetes duration and glycated hemoglobin (HbA_1c_) (model 3).

**Results:**

Median follow-up was 4.7 years and 248 patients (191 men vs. 57 women) experienced the composite end-point. Patients with high EAT (> median level) had increased risk of the composite end-point in model 1 [Hazard ratio (HR): 1.46 (1.13; 1.88), *p *= 0.004], model 2 [HR: 1.31 (1.01; 1.69), *p *= 0.038], and borderline in model 3 [HR: 1.32 (0.99; 1.77), *p *= 0.058]. For men, but not women, high EAT was associated with a 41% increased risk of CVD and mortality in model 3 (*p *= 0.041). Net reclassification index improved when high EAT was added to model 3 (19.6%, *p *= 0.035). PAT or CAT were not associated with the end-point.

**Conclusion:**

High levels of EAT were associated with the composite of incident CVD and mortality in patients with type 2 diabetes, particularly in men, after adjusting for CVD risk factors. EAT modestly improved risk prediction over CVD risk factors.

## Background

Despite aggressive treatment regimens and improved diagnostics, patients with type 2 diabetes remain at a substantially higher risk of cardiovascular complications compared to patients without diabetes [1]. This highlights the need for novel biomarkers that can improve early identification of patients at risk of cardiovascular disease (CVD). In recent years, cardiac fat [including epicardial (EAT) and pericardial (PAT) fat] has gained attention as a promising imaging biomarker of coronary artery disease (CAD) due to its intimate relation to the myocardium and coronary arteries [2]. A recent meta-analysis of 13 studies reported that patients with type 2 diabetes have more cardiac fat compared to individuals without diabetes [3]. Although, the association of EAT and CVD is well established in both community-based populations and in patients with CAD [4–11], evidence from patients with type 2 diabetes is still sparse [12]. Moreover, it remains uncertain if the association of EAT and CVD is truly independent from traditional risk factors in patients with type 2 diabetes. A few studies show that EAT provides incremental value to existing risk factors in the prediction of future CVD in the general population and in patients with stable chest pain [13, 14]. However, the clinical relevance, as assessed by improvement in risk prediction, of adding EAT, PAT and total cardiac fat (CAT) in the prediction of incident CVD and mortality has not been addressed in patients with type 2 diabetes. Moreover, it is not known whether there are sex-specific differences in the pathogenicity of cardiac fat in type 2 diabetes.

Sex is one of the traditional risk factors of CAD. Men are at a higher risk compared to women of similar age [15] due to a different risk profile, which includes a larger amount of android/visceral adipose tissue in men compared to a preferential gynoid fat distribution in women [16, 17]. The physiology of these adipose tissue depots differs: while gynoid adipose tissue is protective, android and visceral adipose tissues are proinflammatory, metabolically active and prone to hypertrophy, which are traits believed to convey the cardiometabolic pathogenicity of visceral fat [18–20]. EAT holds similar pathogenic characteristics with a pro-inflammatory secretory capacity [21] and a high lipolytic activity which exceeds that of intra-abdominal fat [22]. Men are known to have more cardiac fat compared to women [23], yet, it is not known whether this translates to an increased risk of CVD.

The aim of this study was to investigate whether cardiac fat (CAT, EAT, PAT) could add to the prognostic value of traditional risk factors in the prediction of incident CVD and mortality in a cohort of patients with type 2 diabetes followed at specialized diabetes clinics. The hypothesis was that cardiac fat improves predictive potential of incident CVD and mortality when added to traditional CVD risk factors. Additionally, we tested for sex differences in the predictive potential of cardiac fat.

## Methods

### Study population

The Thousand & 2 study is a prospective cohort study initiated in 2011. Patients were recruited from Steno Diabetes Center Copenhagen and Center for Diabetes Research, Gentofte Hospital [24–26] and all patients with type 2 diabetes followed at either center were eligible to participate. In total, 2158 patients with type 2 diabetes were invited and 1030 participated. At inclusion patients filled out a questionnaire with information about current medication, history of coronary heart disease (myocardial infarction, percutaneous coronary intervention, coronary artery bypass grafting, congestive heart failure, and atrial fibrillation), and information about cardiovascular risk factors (prior stroke, peripheral artery disease, family history of CAD, and smoking status). At the study visit, routine blood samples using standard hospital assays were retrieved (cholesterol, high-density lipoprotein (HDL), low-density lipoprotein (LDL), triglyceride, glycated hemoglobin (HbA_1c_), and albuminuria) as described previously [24, 27]. Body mass index (BMI) was calculated as weight divided by the squared height.

### Echocardiographic measurement of cardiac adipose tissue

EAT thickness was measured as the echo-free space above the free wall of the right ventricle to the epicardium at end-systole in the parasternal long axis view perpendicular to the aortic annulus [28]. PAT thickness was measured as the hypoechoic space in front of EAT and on the external pericardium [28]. EAT and PAT were combined to obtain CAT. The CAT measurements have been previously validated in our group by assessment of intra- (0.93) and inter-observer (0.96) correlation coefficients [12].

### Follow-up

Follow-up was performed after 4.7 [median, interquartile range (IQR) 4.0; 5.3] years using ‘The Danish National Board of Health’s National Patient Registry and Registry of Cause of Death’ as described previously [29]. The composite end-point comprised incident non-fatal CVD events (coronary revascularization, myocardial infarction, heart failure, cardiac arrest, cerebrovascular disease, and peripheral artery disease) and all-cause mortality. Follow-up was 99.9%, as one person was lost to follow-up because of incorrectly collected social security number.

### Statistics

Statistical analyses were performed using SPSS (version 20.0, Chicago, Illinois, USA), SAS (version 9.3, SAS Institute, Cary, North Carolina, USA) and R for Mac, version 3.4.3 (R Project for Statistical Computing, Vienna University of Economics and Business Administration, Wien, Austria). Continuous traits were reported as mean ± standard deviation (SD) or as median and interquartile range (IQR) when not normally distributed. Categorical data are shown as numbers (n) and percentages (%). Continuous variables were compared using the pooled 2-sample t-tests or Mann–Whitney U tests as appropriate. Categorical variables were compared using Chi-square tests. EAT, PAT and CAT were analyzed both as continuous and as binary (split by the median fat level) variables. Cox proportional hazard regression models were performed to obtain hazard ratios (HRs) with 95% confidence intervals (CIs) for the risk of the composite end-point per mm increment of cardiac fat. Model 1 was unadjusted. Model 2 was adjusted for age and sex. Model 3 was adjusted for CVD risk factors including age, sex, systolic blood pressure, LDL, BMI, smoking, diabetes duration and HbA_1c_. Non-linearity was tested by adding cardiac fat squared as a covariate to the models. The interaction of sex and cardiac fat on the risk of the composite end-point was analyzed in model 1–3. Propensity score matching of males and females was performed to eliminate differences in confounding baseline characteristics (age, systolic blood pressure, LDL, BMI, smoking, diabetes duration and HbA_1c_), and the resulting matched study population was analyzed for association of EAT and the composite end-point in males vs. females, respectively. The additive predictive value of EAT, PAT and CAT was tested with C-statistics and the net reclassification index (NRI) [30]. A two-tailed p < 0.05 was considered statistically significant.

## Results

### Study population characteristics

Median follow-up time was 4.7 (IQR: 4.0; 5.3) years for the composite end-point of incident CVD and mortality. Of the entire population 248 (24%) experienced a composite end-point. More men experienced the composite end-point compared to women [191 (28%) vs. 57 (16%), *p *< 0.001]. Women had higher LDL and higher total cholesterol, and there was no sex-difference in other CVD risk factors assessed including age, smoking, diabetes duration and BMI (Table 1). Men had higher levels of CAT [12.1 (SD: 3.7) mm vs. 11.4 (3.5), *p *= 0.004] and PAT [7.1 (2.4) vs. 6.2 (2.6), *p *< 0.001] compared to women. EAT was not different in men vs. women [5.0 (1.8) vs. 5.0 (1.9), *p *= 0.86] (Table 1).

**Table 1:** Baseline characteristics

Characteristic	Total	Men	Women	*p* value
Participants, n (%)	1030	679 (66)	351 (34)	**<** ***0.001***
Age, years	65 (10)	65 (10)	64 (11)	*0.81*
Current smoker, (%)	151 (15)	105 (16)	46 (13)	*0.31*
Diabetes duration, years	11 [6, 17]	12 [6, 17]	10 [5, 17]	*0.19*
Ischemic heart disease, n (%)	192 (19)	161 (24)	31 (8)	**<** ***0.001***
Systolic blood pressure (mmHg)	136 (17)	134 (18)	138 (17)	***0.001***
Body Mass Index (kg/m^2^)	30.3 (5.7)	30.1 (5.1)	30.6 (6.6)	*0.16*
Cholesterol (mmol/l)	4.1 [3.5; 4.8]	4.0 [3.4; 4.6]	4.4 [3.8; 5.1]	**<** ***0.001***
LDL (mmol/l)	2.0 [1.2; 1.8]	1.9 [1.5; 2.5]	2.1 [1.6; 2.8]	***0.001***
HDL (mmol/l)	1.2 [1.0; 1.4]	1.1 [0.9; 1.3]	1.3 [1.1; 1.6]	**<** ***0.001***
Triglyceride (mmol/l)	1.8 [1.2; 2.5]	1.8 [1.2; 2.6]	1.6 [1.2; 2.3]	***0.032***
HbA_1C_ (mmol/mol)	55 [48; 66]	55 [48; 65]	54 [46; 66]	*0.69*
P-creatinine (µmol/l)	79 [66; 98]	84 [73; 104]	67 [56; 83]	**<** ***0.001***
CAT (mm)	11.9 (3.6)	12.1 (3.7)	11.4 (3.5)	***0.004***
EAT (mm)	5.0 (1.8)	5.0 (1.8)	5.0 (1.9)	*0.86*
PAT (mm)	6.9 (2.8)	7.1 (2.8)	6.4 (2.6)	**<** ***0.001***
Composite end-point, n (%)	248 (24)	191 (28)	57 (16)	**<** ***0.001***

Data is expressed as means (SD), medians (IQR) and number (n) and percentage (%). Pooled 2-sample t-tests was used for parametric means (SD) or Mann–Whitney U tests for non-parametric data (median (IQR) values). Chi^2^ test was used for categorical data

*EAT* epicardial adipose tissue, *HbA*_*1c*_ glycated hemoglobin, *HDL* high-density lipoprotein, *LDL* low-density lipoprotein, *PAT* pericardial adipose tissue, *CAT* total cardiac adipose tissue

### Epicardial adipose tissue and cardiovascular disease and all-cause mortality

**Fig. 1:**
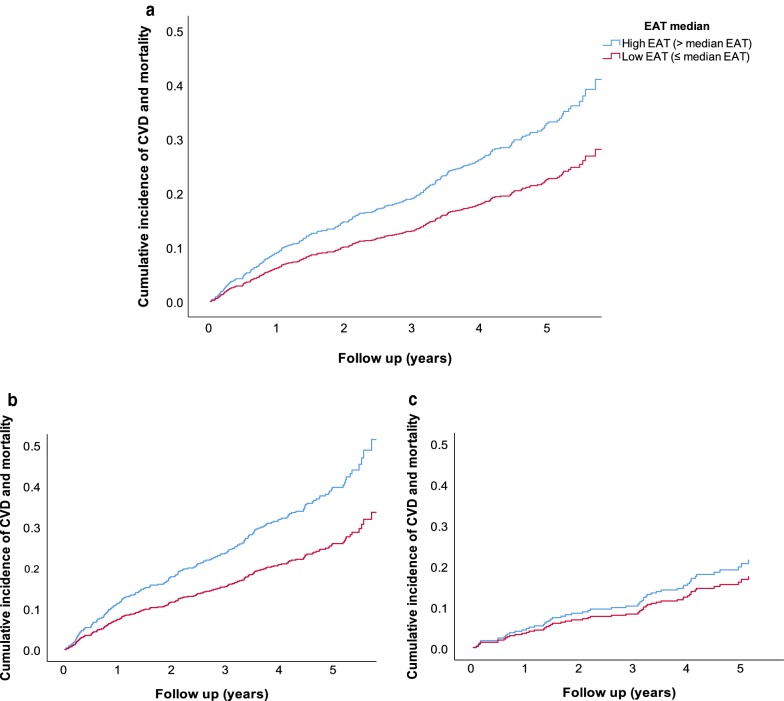
Association of high vs. low EAT to risk of CVD and all-cause mortality. Kaplan–Meyer proportional hazards plot of epicardial adipose tissue (EAT) and risk of the composite end-point CVD or mortality in patients with EAT levels below the median (low EAT) group (blue) and above the median (high EAT) group (green). Hazard ratio (HR) 1.46 confidence interval (CI) 1.13; 1.88, *p *= 0.004 in the total population (**a**), HR 1.54 confidence interval (CI) 1.15; 2.10, *p *= 0.004 in men (**b**) and in women (**c**) HR: 1.22, CI 0.72; 2.06, *p *= 0.46

EAT as a continuous variable was borderline associated with the composite end-point of incident CVD and mortality in the unadjusted model 1 [HR: 1.06 (1.00; 1.14), *p *= 0.057], and the association was significant in men [HR: 1.08 (1.01; 1.17), *p *= 0.033], but not in women [HR: 1.02 (0.89; 1.17), *p *= 0.76] (Additional file 1: Table S1). The interaction of sex and EAT on the composite end-point was, however, not significant (*p *= 0.44). The association in men did not remain significant in model 2 [HR: 1.05 (0.98; 1.13), *p *= 0.18] or after further adjustment (Additional file 1: Table S1). EAT was not associated with the composite end-point in a non-linear manner [HR: 1.00 (0.99; 1.00), *p *= 0.55]. In analyses where EAT was dichotomized by the median [4.6 (IQR: 3.8; 5.8) mm], patients with high EAT levels (above the median) had an increased risk of the composite end-point in model 1 [HR: 1.46 (1.13; 1.88), *p *= 0.004], and the association was significant in men [HR: 1.54 (1.15; 2.10), *p *= 0.004], but not in women [HR: 1.22 (0.72; 2.06), *p *= 0.46] (Fig. 1). The association remained significant in the total population in model 2 [HR: 1.31 (1.01; 1.69), *p *= 0.038], and was borderline significant in model 3 [HR: 1.32 (0.99; 1.78), *p *= 0.057]. For male patients, high EAT levels remained significantly associated with an increased risk of incident CVD and mortality in model 3 [HR: 1.41 (1.01; 1.96), *p *= 0.041] (Table 2). Using the propensity score matched study population (n = 534, 50% males/females) resulted in similar findings, where high EAT remained associated with an increased risk of incident CVD and mortality in the total population [HR: 1.54 (1.06; 2.23), *p *= 0.022] and a tendency was found in men [1.49 (0.94; 2.36), *p *= 0.093] but not in women [1.44 (0.77; 2.68), *p *= 0.25]. No significant interaction was observed between high EAT levels and sex on the composite end-point in model 3 (*p *= 0.50) or in the propensity score matched population (*p *= 0.95).

**Table 2:** Association of high levels of cardiac fat (> median) with CVD and all-cause mortality

Adipose tissue	Model	Composite (n = 248)		Men		Women	
				Composite (n = 191)		Composite (n = 57)	
		HR (95% CI)	*p* value	HR (95% CI)	*p* value	HR (95% CI)	*p* value
High EAT	1	1.46 (1.13; 1.88)	***0.004***	1.54 (1.15; 2.10)	***0.004***	1.22 (0.72; 2.06)	*0.46*
	2	1.31 (1.01; 1.69)	***0.038***	1.40 (1.05; 1.87)	***0.024***	1.08 (0.63; 1.83)	*0.79*
	3	1.32 (0.99; 1.78)	*0.057*	1.41 (1.01; 1.96)	***0.041***	1.16 (0.62; 2.17)	*0.65*
High PAT	1	1.07 (0.83; 1.37)	*0.62*	0.95 (0.72; 1.27)	*0.46*	1.22 (0.72; 2.07)	*0.46*
	2	0.90 (0.70; 1.16)	*0.42*	0.83 (0.63; 1.10)	*0.21*	1.15 (0.67; 1.95)	*0.62*
	3	0.84 (0.63; 1.12)	*0.23*	0.79 (0.57; 1.09)	*0.16*	1.05 (0.56; 1.97)	*0.87*
High CAT	1	1.20 (0.93; 1.54)	*0.16*	1.14 (0.86; 1.52)	*0.43*	1.24 (0.73; 2.10)	*0.43*
	2	1.02 (0.79; 1.31)	*0.88*	1.00 (0.75; 1.33)	*0.99*	1.10 (0.64; 1.87)	*0.73*
	3	0.97 (0.72; 1.30)	*0.82*	0.97 (0.69; 1.35)	*0.86*	1.04 (0.55; 1.98)	*0.62*

HR values are presented according to high (> median) vs. low (< median) levels of adipose tissue

Model 1 is unadjusted

Model 2 is adjusted for age, sex

Model 3 is adjusted for age, sex, LDL, diabetes duration, HbA_1c_, systolic blood pressure, smoking and BMI

*CI* confidence interval, *EAT* epicardial adipose tissue, *HR* hazard ratio, *PAT* pericardial adipose tissue, *CAT* total cardiac adipose tissue

While high EAT levels were associated with both mortality [1.61 (1.09; 2.38), *p *= 0.018] and incident CVD [1.41 (1.00; 1.97), *p *= 0.046] in the male population in model 1, no significant association with either mortality [2.00 (0.84; 4.50), *p *= 0.09] or incident CVD [1.12 (0.58; 2.18), *p *= 0.74] was found for females (Additional file 2: Table S2).

### Pericardial adipose tissue and cardiovascular disease and all-cause mortality

PAT was not associated with the composite end-point in model 1–3, and, in contrast to EAT, sex did not modify the association (Additional file 1: Table S1). PAT was not associated with the composite end-point in a non-linear manner [HR: 0.99 (0.98; 1.00), *p *= 0.31]. The median level of PAT was 6.3 (IQR: 4.8; 8.6) mm, and patients with high levels of PAT (above the median) did not have an increased risk of the composite end-point in model 1–3 and sex did not modify the result (Table 3). No association was found of high PAT with either mortality or incident CVD in model 1–3 (Additional file 2: Table S2).

**Table 3:** Performance of adipose tissue in predicting the composite end-point

All patients	C-statistics		Net reclassification index	
	Model 1 (95% CI)	Model 3 (95% CI)	Model without vs. with adipose tissue	
			Percent (95% CI)	*p* value
Model *without* adipose tissue		0.67 (0.63–0.72)		
Model including				
EAT	0.53 (0.49–0.58)	0.68 (0.63–0.72)	19.6% (1.4–37.8)	0.035
High EAT	0.54 (0.50–0.58)	0.67 (0.63–0.72)	20.8% (2.5–39.1)	0.026
Men				
Model *without* adipose tissue		0.65 (0.60–0.70)		
Model including				
EAT	0.54 (0.49–0.59)	0.66 (0.61–0.70)	17.7% (− 3.1 to 38.5)	0.09
High EAT	0.55 (0.50–0.59)	0.65 (0.60–0.70)	19.6% (1.1–40.0)	0.06
Women				
Model *without* adipose tissue		0.66 (0.55–0.76)		
Model including				
EAT	0.50 (0.39–0.60)	0.66 (0.56–0.77)	− 4.3% (− 42.8 to 34.1)	0.83
High EAT	0.52 (0.43–0.60)	0.66 (0.55–0.77)	20.3% (20.6–61.2)	0.33

Adipose tissue variables are presented as continuous or as binary variables: high (> median) vs. low (< median) levels of adipose tissue

Model 1 is unadjusted

Model 3 is adjusted for age, sex, LDL, diabetes duration, HbA_1C_, systolic blood pressure, smoking and BMI

*CI* confidence interval, *EAT* epicardial adipose tissue, *HR* hazard ratio, *PAT* pericardial adipose tissue, *CAT* total cardiac adipose tissue

### Total cardiac adipose tissue and cardiovascular disease and all cause-mortality

CAT was associated with increased risk of the composite end-point of incident CVD and mortality in model 1 [HR: 1.04 (1.00; 1.07), *p *= 0.035] but not when stratified into men and women (Additional file 1: Table S1). The association did not remain significant in model 2–3 or when the population was stratified by sex (Additional file 1: Table S1). CAT was not associated non-linearly with the composite end-point [HR: 1.00 (0.97; 1.01), *p *= 0.44]. Patients with CAT levels above the median [11.5 (IQR: 9.2; 14.3) mm] did not have an increased risk of the composite end-point in model 1–3 (Table 2). No association was found of high CAT with either mortality or incident CVD in model 1–3 (Additional file 2: Table S2).

### Predictive value of cardiac adipose tissue in the risk assessment of cardiovascular disease and all-cause mortality

When examining model performances using C-statistic we found similar results before and after adding EAT (both as a continuous and binary variable) in both unadjusted (model 1) and adjusted (model 3) analyses (Table 3). We found that in both model 1 and model 3, high EAT (above the median) and EAT (as a continuous variable) modestly improved predictive model performance using net reclassification index (Table 3). Addition of EAT or high EAT to model 3 correctly reclassified a large proportion of the total population [19.6%, *p *= 0.035 or 20.5%, *p *= 0.026, respectively] (Table 3). Additionally, in men the same trend was found although insignificantly (Table 3). In women, EAT or high EAT did not improve the net classification index.

## Discussion

In this prospective study of 1030 patients with type 2 diabetes, we found that high levels of epicardial adipose tissue levels (above the median) were associated with the composite end-point of incident CVD and mortality after 4.7 years of follow-up. There was, however, no evidence that epicardial adipose tissue, as a continuous variable, was associated with the composite end-point. When stratified by sex, the association of high epicardial adipose tissue and risk of incident CVD and mortality remained for men, even after further adjustment for CVD risk factors (age, BMI, LDL, systolic blood pressure, smoking, diabetes duration, and HbA_1c_) indicating a potential novel sexual dimorphism of the pathogenicity of EAT, which, however, needs confirmation in future studies. Epicardial adipose tissue (both as a continuous and binary variable) when added to the adjusted model (model 3) modestly improved predictive value in the assessment of risk of incident CVD and mortality and only when examined by net reclassification index.

### Cardiac fat and prediction of CVD complications in patients with type 2 diabetes

In the search of novel biomarkers that could improve identification of patients with type 2 diabetes at risk of CVD, EAT as an imaging biomarker has gained attention due to its intimate location to the coronary vessels and the myocardium [2, 31]. A recent meta-analysis has provided evidence that EAT is associated with prevalent subclinical atherosclerosis, ischemia, and future major adverse cardiac events [32], but only a few longitudinal studies have evaluated the predictive potential of EAT as an imaging biomarker of future CVD. The Multi-Ethnic Study of Atherosclerosis (MESA) reported that CAT was associated with increased risk of coronary heart disease (CHD) in a community-based case–control study of 998 controls and 147 cases that developed CHD over a 5 year period [4]. Cheng et al. [5] confirmed these findings in patients with asymptomatic CHD, and furthermore found a trend that EAT could improve MACE prediction when added to the Framingham Risk Score and coronary calcium scoring (CCS). Following these findings, more longitudinal studies unanimously concluded that high levels of EAT were associated with increased risk of CVD in patients with known or suspected CHD [8–10, 14]. A recent study showed that EAT volume is higher and density lower in subjects with coronary calcium compared to subjects without, and that EAT was more significantly associated with MACE than the calcium score [33]. Despite the solid evidence of EAT and CVD in general, evidence from patients with type 2 diabetes is still sparse and the predictive potential of EAT in type 2 diabetes has not been determined. Our group has previously shown for high-risk patients with type 2 diabetes and albuminuria that high-levels of cardiac fat were associated with increased risk of incident CVD or mortality in a 5-year follow-up study [12]. This study extends these findings to an unselected cohort of patients with type 2 diabetes.

The next step is to determine the clinical relevance, establish threshold values, and evaluate whether adding cardiac fat to existing cardiac risk algorithms improves prognostic performance. To this end a recent paper by Zhou et al. demonstrated that prediction of obstructive CAD was improved when EAT > 100 ml (as a binary marker) was added to a model based on age, sex, angina, and calcium score in patients with stable angina [13]. In the present study, we demonstrate for the first time in patients with type 2 diabetes a modest improvement in the prediction of incident CVD and mortality when EAT (both as a binary and continuous marker) was added to a model including age, sex, BMI, smoking, LDL cholesterol, and diabetes duration evaluated using net reclassification index. Conversely, C-statistic is relatively insensitive at comparing models after inclusion of CVD risk factors [34], which may explain the lack of improvement in model performance when evaluated by this method.

Despite, EAT, PAT, and CAT have been independently associated with increased risk of CVD [4, 5, 10] their individual importance needs to be determined. In our study PAT was not associated with increased risk of incident CVD and mortality. Moreover, the physiological status of EAT (e.g. inflamed vs. non-inflamed EAT) rather than EAT mass may be a superior measure of cardiac risk, as suggested by Oikonomou et al., who revealed in the CRISP-CT study that inflammation-induced changes in perivascular EAT assessed by fat attenuation on computer tomography angiography (CTA) enhanced risk prediction substantially above traditional risk factors [35]. Thus, more studies are warranted to determine the clinical relevance of EAT, and to determine whether adding EAT mass or functional properties (e.g. inflammation) will be superior as cardiac risk measures.

### Sex-differences in adipose tissue distribution and CVD risk

Men have an android and visceral fat distribution compared to women, and it is well known that both the amount but also the physiological properties (e.g. secretion of inflammatory adipokines) of visceral fat are detrimental to cardiometabolic health [18–20]. Patients with visceral obesity and middle-aged subjects with suspected metabolic syndrome have higher levels of epicardial adipose tissue thickness, which was moreover, associated with cardiac fibrosis development 1-year post-myocardial infarction [36]. Therefore, the present finding that men have more CAT might offer a contributing explanation as to why men are at a higher risk of CHD compared to women of similar age [15]. Curiously, in our study EAT levels were not different in men and women, but high EAT (> median) was only associated with increased risk of the composite end-point in men and not women. A recent study suggest this to be due to an increased pathogenicity (e.g. increased inflammation) of EAT in men vs. women since EAT only in men was associated with systemic inflammation represented by high-sensitive CRP levels, increased left ventricular mass, and subclinical myocardial dysfunction [37]. Another study supports a sexual dimorphism of the association of EAT and inflammatory markers (e.g. IL-6) as this was only found in male and not female rats [38]. Although the sex-specific association with hard CVD end-points, as shown in this study, is novel, it will need confirmation in larger trials, as we cannot exclude that the lack of association in women merely reflects the lower event rate in this population reducing overall power of the associations.

### Clinical relevance of cardiac fat

The last decades of research in cardiac fat has shown EAT to be a promising novel imaging biomarker of cardiovascular risk in patients with suspected or known CHD. Now important papers have emerged where EAT/EAT attenuation measured by CTA improves the predictive potential of existing cardiac risk models in patients at risk/with CHD [13, 35].

EAT is a transducer of the adverse effects of systemic inflammation and metabolic disorders on the heart, and thus, represents an important target for therapeutic interventions [39–41]. In patients with type 2 diabetes, there was recently reported a positive association of EAT and severity of coronary artery disease [42]. The present study extends this finding by demonstrating that EAT measured by echocardiography modestly improved risk prediction in patients with type 2 diabetes. Regarding pharmacological influences, EAT thickness at systole is a consistent independent predictor of new onset diabetes in patients with CAD treated with high-intensity statins [43]. Treatment with dapagliflozin or non-pharmacological exercise-based interventions might be means to improve systemic metabolic parameters and decrease the EAT volume in patients with/at risk of diabetes [44, 45].

Yet, more work is needed to fully establish the role of EAT in cardiac risk prediction and as a therapeutic target in asymptomatic patients with type 2 diabetes at with/at risk of CVD where CTA is not ruinously performed, and echocardiography is the initial option for EAT evaluation.

### Limitations

Even though a strength of this study is its large scale where 1030 patients with type 2 diabetes were included, the study has some limitations. First, cardiac fat was measured by echocardiography and not by the gold-standard magnetic resonance imaging or CTA limiting the conclusions of the study. However, cardiac fat thickness measured by echocardiography has been shown to correlate with volumetric measurements [46]. Second, since we address sex differences in the predictive ability of cardiac fat, other sex-specific anthropometric measures of fat distribution (waist and hip circumference, android, gynoid, and visceral fat mass) would be highly relevant. Unfortunately, we did not have access to these measures, but we did account for BMI in our model allowing us to address the independent effect of cardiac fat without the contribution of overall adiposity. Third, even though the population was large and allowed for subgroup analysis the event rate was smaller in the female population compared to the male population, which could reduce statistical power in the sex-divided subgroup analysis thus limiting the conclusion. We did not find a significant interaction between sex and high EAT, which further limits the conclusion and suggests that the sex difference could be due to differences in sample size or baseline characteristics. To eliminate these differences between females and males the analysis was repeated in the propensity score matched study population. Results from this analysis indicated a sex difference in the association of high EAT and the composite end-point, although replication of these findings are needed in future studies.

## Conclusion

In this study in patients with type 2 diabetes, we found that high levels of epicardial adipose tissue (above the median) was associated with increased risk of the composite end-point of incident CVD and mortality, particularly in men, after 4.7 years of follow-up. High levels of EAT modestly improved predictive potential of cardiac risk when added to a model including available traditional CVD risk factors. Future studies are warranted to elucidate whether EAT has a role in the formal prognostic clinical algorithms.

## Supplementary information

**Additional file 1: Table S1.** Association of cardiac fat with CVD and all-cause mortality: HR values are presented per mm increase in adipose tissue thickness. Abbreviations: confidence interval (CI), epicardial adipose tissue (EAT), hazard ratio (HR), pericardial adipose tissue (PAT), total cardiac adipose tissue (CAT). Model 1 is unadjusted. Model 2 is adjusted for age, sex. Model 3 is adjusted for age, sex, LDL, diabetes duration, HbA_1c_, systolic blood pressure, smoking and BMI.

**Additional file 2: Table S2.** Association of high cardiac fat (> median) with CVD or all-cause mortality. Abbreviations: confidence interval (CI), epicardial adipose tissue (EAT), hazard ratio (HR), pericardial adipose tissue (PAT), total cardiac adipose tissue (CAT). Model 1 is unadjusted. Model 2 is adjusted for age. Model 3 is adjusted for age, LDL, diabetes duration, HbA_1c_, systolic blood pressure, smoking and BMI.

## Data Availability

The datasets generated during and/or analyzed during the current study are available from the corresponding author and the senior author on reasonable request.

## Abbreviations

BMI: body mass index
CAD: coronary artery disease
CAT: total cardiac adipose tissue (i.e. EAT + PAT)
CCS: coronary calcium score
CHD: coronary heart disease
CI: confidence intervals
CVD: cardiovascular disease
CTA: computer tomography angiography
EAT: epicardial adipose tissue
HbA_1c_: glycated hemoglobin
HDL: high-density lipoprotein
HR: hazard ratio
IQR: interquartile range
LDL: low-density lipoprotein
NRI: net reclassification index
PAT: pericardial adipose tissue
