# Supplementary material for: Epicardial adipose tissue predicts incident cardiovascular disease and mortality in patients with type 2 diabetes
Source: Cardiovasc Diabetol. 2019 Aug 30;18:114. doi: 10.1186/s12933-019-0917-y (PMC6716926; doi:10.1186/s12933-019-0917-y)
Supplement: Supplementary file 1 — Additional file 1: Table S1. Association of cardiac fat with CVD and all-cause mortality: HR values are presented per mm increase in adipose tissue thickness. Abbreviations: confidence interval (CI), epicardial adipose tissue (EAT), hazard ratio (HR), pericardial adipose tissue (PAT), total cardiac adipose tissue (CAT). Model 1 is unadjusted. Model 2 is adjusted for age, sex. Model 3 is adjusted for age, sex, LDL, diabetes duration, HbA1c, systolic blood pressure, smoking and BMI. [file 12933_2019_917_MOESM1_ESM.docx]

**Table S1: Association of cardiac fat with CVD and all-cause mortality**

|  |  |  | | Men | | Women | | |
| --- | --- | --- | --- | --- | --- | --- | --- | --- |
| Adipose tissue | Model | Composite (n=248) | | Composite (n=191) | | Composite (n=57) | | |
|  |  | HR (95% CI) | *p* value | HR (95% CI) | *p* value | | HR (95% CI) | *p* value |
| EAT | 1 | 1.06 (1.00;1.14) | *0.057* | 1.08 (1.01;1.17) | ***0.033*** | | 1.02 (0.89;1.17) | *0.76* |
|  | 2 | 1.03 (0.97;1.10) | *0.31* | 1.05 (0.98;1.13) | *0.18* | | 0.99 (0.86;1.14) | *0.84* |
|  | 3 | 1.05 (0.98;1.13) | *0.18* | 1.07 (0.98;1.16) | *0.13* | | 1.02 (0.87;1.21) | *0.80* |
| PAT | 1 | 1.03 (0.99;1.08) | *0.15* | 1.01 (0.96;1.07) | *0.68* | | 1.06 (0.96;1.17) | *0.26* |
|  | 2 | 1.00 (0.96;1.05) | *0.87* | 0.99 (0.95;1.05) | *0.83* | | 1.04 (0.94;1.15) | *0.43* |
|  | 3 | 0.98 (0.93;1.04) | *0.55* | 0.98 (0.92;1.03) | *0.40* | | 1.03 (0.91;1.16) | *0.67* |
| CAT | 1 | 1.04 (1.00;1.07) | ***0.035*** | 1.03 (0.99;1.07) | *0.18* | | 1.04 (0.97;1.12) | *0.27* |
|  | 2 | 1.01 (0.98;1.05) | *0.48* | 1.01 (0.97;1.05) | *0.59* | | 1.02 (0.95;1.10) | *0.57* |
|  | 3 | 1.00 (0.97;1.05) | *0.85* | 1.00 (0.96;1.05) | *0.93* | | 1.07 (0.80;1.12) | *0.64* |

HR values are presented per mm increase in adipose tissue thickness. Abbreviations: confidence interval (CI), epicardial adipose tissue (EAT), hazard ratio (HR), pericardial adipose tissue (PAT), total cardiac adipose tissue (CAT).

Model 1 is unadjusted.

Model 2 is adjusted for age, sex.

Model 3 is adjusted for age, sex, LDL, diabetes duration, HbA_1c_, systolic blood pressure, smoking and BMI.
