# Supplementary material for: Epicardial adipose tissue predicts incident cardiovascular disease and mortality in patients with type 2 diabetes
Source: Cardiovasc Diabetol. 2019 Aug 30;18:114. doi: 10.1186/s12933-019-0917-y (PMC6716926; doi:10.1186/s12933-019-0917-y)
Supplement: Supplementary file 2 — Additional file 2: Table S2. Association of high cardiac fat (> median) with CVD or all-cause mortality. Abbreviations: confidence interval (CI), epicardial adipose tissue (EAT), hazard ratio (HR), pericardial adipose tissue (PAT), total cardiac adipose tissue (CAT). Model 1 is unadjusted. Model 2 is adjusted for age. Model 3 is adjusted for age, LDL, diabetes duration, HbA1c, systolic blood pressure, smoking and BMI. [file 12933_2019_917_MOESM2_ESM.docx]

**Table S2: Association of high cardiac fat (> median fat) with CVD or all-cause mortality**

|  |  | Men | | Women | | Men | | Women | |
| --- | --- | --- | --- | --- | --- | --- | --- | --- | --- |
| Cardiac Fat | Model | Mortality (n=105) | | Mortality (n=26) | | Incident CVD (n=138) | | Incident CVD (n=36) | |
|  |  | HR (95% CI) | *p* value | HR (95% CI) | *P* value | HR (95% CI) | *p* value | HR (95% CI) | *p* value |
| High EAT | 1 | 1.61 (1.09;2.38) | ***0.018*** | 2.00 (0.84;4.50) | 0.09 | 1.41 (1.00;1.97) | ***0.046*** | 1.12 (0.58;2.18) | 0.74 |
|  | 2 | 1.45 (0.98;2.15) | *0.067* | 1.72 (0.76;3.90) | 0.20 | 1.28 (0.91;1.80) | 0.15 | 0.98 (0.50;1.93) | 0.96 |
|  | 3 | 1.28 (0.83;1.99) | *0.27* | 1.33 (0.52;3.36) | 0.55 | 1.31 (0.89;1.93) | 0.17 | 1.24 (0.55;2.80) | 0.60 |
| High PAT | 1 | 0.96 (0.65;1.42) | *0.84* | 1.25 (0.57;2.73) | 0.58 | 0.89 (0.64;1.24) | 0.89 | 1.14 (0.59;2.17) | 0.70 |
|  | 2 | 0.85 (0.57;1.25) | *0.40* | 1.16 (0.53;2.53) | 0.72 | 0.78 (0.56;1.09) | 0.15 | 1.08 (0.55;2.09) | 0.83 |
|  | 3 | 0.76 (0.49;1.18) | *0.22* | 1.21 (0.48;3.07) | 0.69 | 0.70 (0.48;1.02) | 0.07 | 1.11 (0.49;2.50) | 0.80 |
| High CAT | 1 | 1.29 (0.87;1.91) | *0.20* | 1.79 (0.80;3.98) | 0.16 | 1.02 (0.73;1.42) | 0.92 | 1.12 (0.58;2.17) | 0.74 |
|  | 2 | 1.13 (0.76;1.67) | *0.54* | 1.54 (0.69;3.45) | 0.29 | 0.89 (0.64;1.25) | 0.51 | 1.00 (0.51;1.95) | 1.00 |
|  | 3 | 0.99 (0.64;1.55) | *0.98* | 1.34 (0.52;3.49) | 0.55 | 0.80 (0.54;1.18) | 0.80 | 1.11 (0.48;2.55) | 0.81 |

Abbreviations: confidence interval (CI), epicardial adipose tissue (EAT), hazard ratio (HR), pericardial adipose tissue (PAT), total cardiac adipose tissue (CAT).

Model 1 is unadjusted.

Model 2 is adjusted for age

Model 3 is adjusted for age, LDL, diabetes duration, HbA_1c_, systolic blood pressure, smoking and BMI.
